# Adhesion molecules and pancreatitis

**DOI:** 10.1007/s00535-018-1500-0

**Published:** 2018-08-23

**Authors:** Takeshi Sato, Wataru Shibata, Shin Maeda

**Affiliations:** 1 Department of Gastroenterology, Yokohama City University Graduate School of Medicine, Fukuura 3-9, Kanazawa-ku, Yokohama, Kanagawa 236-0004 Japan; 2 Division of Translational Research, Advanced Medical Research Center, Yokohama City University, Fukuura 3-9, Kanazawa-ku, Yokohama, Kanagawa 236-0004 Japan

**Keywords:** Adhesion molecule, Adherens junction, Pancreatitis, Tight junction

## Abstract

Acute and chronic pancreatitises are gastrointestinal inflammatory diseases, the incidence of which is increasing worldwide. Most (~ 80%) acute pancreatitis (AP) patients have mild disease, and about 20% have severe disease, which causes multiple organ failure and has a high mortality rate. Chronic pancreatitis (CP) is characterized by chronic inflammation and destruction of normal pancreatic parenchyma, which leads to loss of exocrine and endocrine tissues. Patients with CP also have a higher incidence of pancreatic ductal adenocarcinoma. Although a number of factors are associated with the development and progression of AP and CP, the underlying mechanism is unclear. Adhesion molecules play important roles in cell migration, proliferation, and signal transduction, as well as in development and tissue repair. Loosening of cell–cell adhesion between pancreatic acinar cells and/or endothelial cells increases solute permeability, resulting in interstitial edema, which promotes inflammatory cell migration and disrupts tissue structure. Oxidative stress, which is one of the important pathogenesis of pancreatitis, leads to upregulation of adhesion molecules. Soluble adhesion molecules are reportedly involved in AP. In this review, we focus on the roles of tight junctions (occludin, tricellulin, claudin, junctional adhesion molecule, and zonula occludin), adherens junctions (E-cadherin and p120-, α-, and β-catenin), and other adhesion molecules (selectin and intercellular adhesion molecules) in the progression of AP and CP. Maintaining the normal function of adhesion molecules and preventing their abnormal activation maintain the structure of the pancreas and prevent the development of pancreatitis.

## Introduction

Acute and chronic pancreatitises are common gastrointestinal inflammatory diseases, the incidence of which is increasing worldwide [1, 2]. Acute pancreatitis (AP) is characterized by sudden-onset abdominal pain, back pain, nausea, vomiting, and high fever. Most patients have mild disease that affects only the pancreas and has a mortality rate of 0.8% in Japan. However, about 20% of patients have severe AP, which causes multiple organ failure and has a mortality rate of 10.1% [2, 3]. In contrast, chronic pancreatitis (CP) is characterized by chronic inflammation with fibrosis and infiltration of inflammatory cells and destruction of normal pancreatic parenchyma, which leads to loss of exocrine (acinar) and endocrine (islet) tissues; this condition is basically irreversible. The main symptoms of CP are recurrent abdominal pain, exocrine and endocrine pancreatic insufficiency, which causes maldigestion, malabsorption, and diabetes [4, 5]. Furthermore, patients with CP have a high incidence of pancreatic ductal adenocarcinoma, particularly those with hereditary and tropical pancreatitis [5]. Although a number of factors—including pancreatic duct obstruction, alcoholism, smoking, and mutation of the gene encoding cationic trypsinogen—are associated with the development and progression of AP and CP, the underlying mechanism is unclear.

**Fig. 1:**
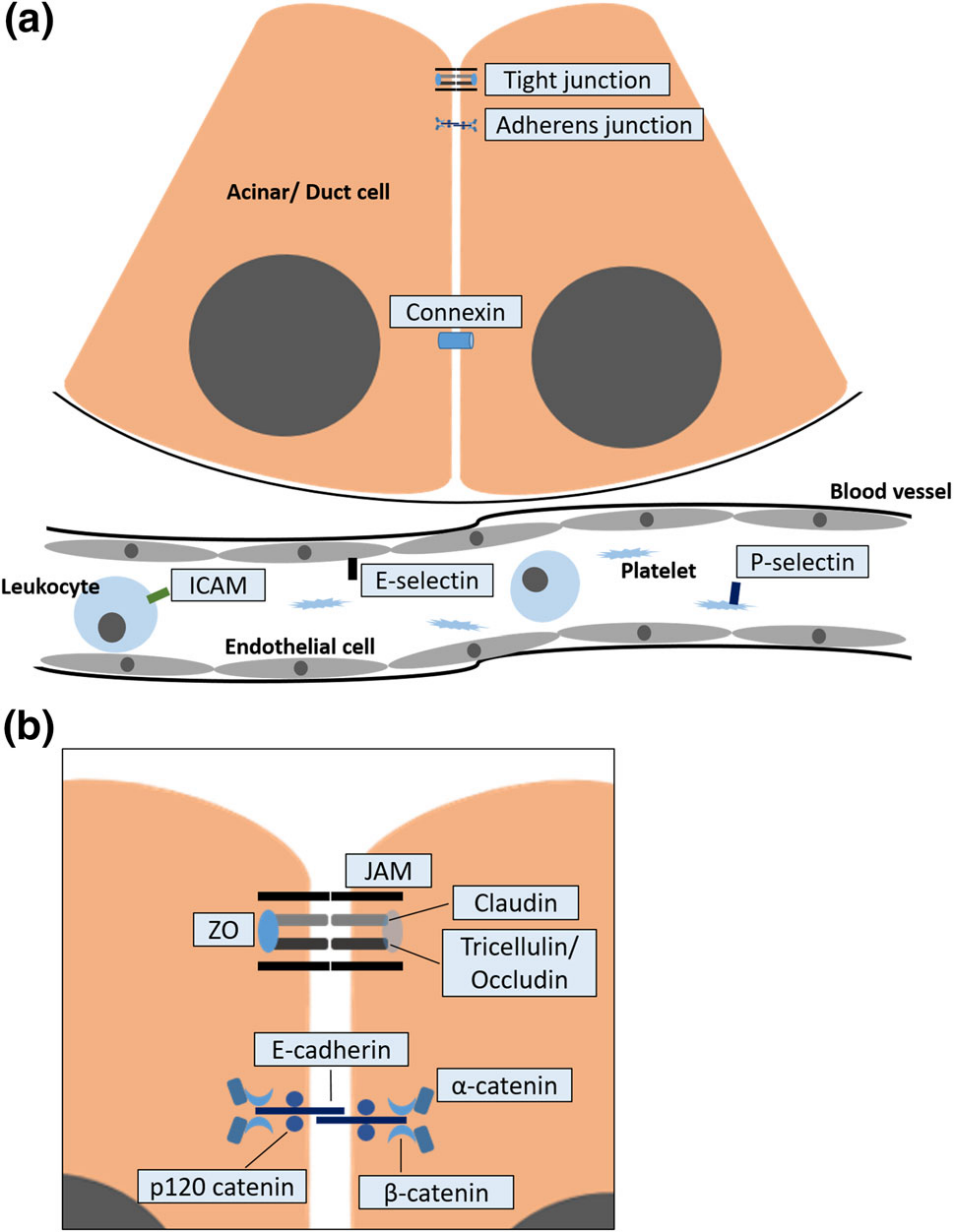
**a** Factors of cell–cell adhesion related to the pathogenesis of AP and CP: tight junctions, adherens junctions, and other adhesion molecules (selectins and ICAMs). **b** Tight junction consists of occludin, tricellulin, claudin, junctional adhesion molecules (JAMs), and zonula occludin (ZO). Adherens junction consists of E-cadherin and p120-, α-, and β-catenin

The histopathological features of AP and CP are structural destruction, infiltration of inflammatory cells, and regeneration with stroma formation and fibrosis; interstitial edema is a characteristic of AP. Adhesion molecules play important roles in cell migration, proliferation, and signal transduction, as well as in development and tissue repair [6, 7]. Loosening of the adhesion between pancreatic acinar cells and/or endothelial cells increases solute permeability, and causes interstitial edema [8]. Overexpression of adhesion molecules, such as intercellular adhesion molecule 1 (ICAM-1), on pancreatic endothelial cells leads to inflammatory cell infiltration into the pancreatic parenchyma [9]. Oxidative stress, which is one of the important pathogenesis of AP and CP, leads to upregulation of adhesion molecules, such as P-selectin and ICAM-1 [10–13]. Moreover, soluble adhesion molecules are also involved in AP, as indicated by increased plasma levels in AP patients [14, 15]. Therefore, adhesion molecules may play important roles in AP and CP. Here, we review the major modes and factors of cell–cell adhesion related to the pathogenesis of AP and CP: tight junctions [occludin, tricellulin, claudin, junctional adhesion molecules (JAMs), and zonula occludin (ZO)], adherens junctions (E-cadherin and p120-, α-, and β-catenin), and other adhesion molecules (selectins and ICAMs) (Fig. 1, Table 1). Although connexins, which form gap junction channels, play important roles in the coordinated function of exocrine and endocrine pancreatic cells [16, 17], their role in pancreatitis is unclear [18]; thus, we omit connexins from this review.

**Table 1:** Expression status and localization of the adhesion molecules in normal pancreas and under pancreatitis conditions

	Normal pancreas	Pancreatitis	
		Pancreas	Other organs
Tight junction			
Occludin	Apical membranes of intralobular duct cells and of acinar cells [25]	Diminished [25]	Distal ileum: upregulated in AP rats [27]
Tricellulin	Apical side of the tricellular contacts of duct cells and of acinar cells [33]	No data	
Claudin			
− 1	Apical membranes of intralobular duct cells and of acinar cells [25]	Disappeared [25]	
− 2	Apical membranes of duct cells [36, 38]	No change in duct cells [38] Upregulated in acinar cells [38]	
− 3	Apical membranes of duct cells and of acinar cells [38]	No change [38]	
− 4	Apical membranes of duct cells and of acinar cells [38]	No change [38]/upregulated in acinar cells [39]	
− 5	Apical membranes of duct cells and of acinar cells [38]	No change [38]	
− 7	Apical membranes of duct cells and of acinar cells [38]	No change [38]	
JAM			
− A	No in vivo data expressed on the membrane of human pancreatic duct epithelial cell line [35]	Upregulated in pancreatic tissue of caerulein AP model [39]	Distal ileum: upregulated in AP rats [27]
− C	Vascular endothelial cells [40]	Upregulated [40]/downregulated [41]	
ZO			
− 1	Apical membranes of intralobular duct cells and of acinar cells [25]	Diminished [25]	Distal ileum: upregulated in AP rats [27]
− 2	No data	No data	
− 3	No data	No data	
Adherens junction			
E-cadherin	Basolateral membranes of duct cells and of acinar cells [8, 52, 53]	Dissociated from the membrane and condensed in the cytosol of acinar cells [8]	
p120 catenin	Basolateral membranes of duct cells and of acinar cells [52, 53]	Decreased protein level in pancreatic tissue of caerulein AP model [46]	
α-Catenin	Basolateral membranes of duct cells and of acinar cells [52, 53]	Decreased protein level in pancreatic tissue of caerulein AP model [46]	
β-Catenin	Basolateral membranes of duct cells and of acinar cells [8, 52, 53]	Dissociated from the membrane and condensed in the cytosol of acinar cells (no localization in the nucleus) [8]	
Other adhesion molecules			
P-Selectin	Focally positive on a few adherent platelets and capillary endothelial cell surfaces [13]	Increase of P-selectin positive adherent platelets [13] Upregulated on the capillary endothelial cell surfaces and postcapillary venules [13]	Lung: upregulated on pulmonary endothelial cells [68, 73]
E-Selectin	Negative [13]	Few positivity on venules [13]	
L-Selectin	No data	No data	
ICAM-1	Week expression in postcapillary venules [13]	Mild increased expression on capillary and postcapillary venular endothelial surfaces [13] Upregulated the membrane-bound ICAM-1 on endothelial cells in experimental AP [9, 83]	

*AP* acute pancreatitis, *JAM* junctional adhesion molecule, *ZO* zonula occludin, *ICAM* intercellular adhesion molecule

### Tight junctions

Tight junctions are the most-apical structures of adjacent cells. Tight junctions are composed of three types of transmembrane protein: occludin/tricellulin, claudin, and JAMs, as well as their associated cytoplasmic ZO proteins. The basic structure and function of these proteins were reviewed by Niessen [19] and Hartsock and Nelson [20]. These proteins function as both a barrier and a fence [19–21]. The barrier function controls the paracellular passage of solutes, ions, and various molecules. The structure of tight junctions in pancreatic exocrine and duct epithelial cells is dynamic and can be disrupted by, for example, carbachol and ductal hypertension [22–24]. Disruption of tight junctions unseals the paracellular spaces of adjacent cells, leading to unlimited movement of solutes, etc., into and out of the paracellular spaces, so causing edema and diarrhea [21]. The fence function maintains cellular polarity to restrict the movement of membrane proteins across the apical and basolateral membranes [19–21]. Disruption of pancreatic epithelial tight junctions is an early event of experimental AP and augments edema in the pancreatic parenchyma [25, 26]. In a rat model, systemic inflammation caused by AP downregulates the expression of tight junction proteins in the intestine, which disrupts the intestinal barrier, leading to bacterial translocation [27]. Thus, tight junctions are likely to play important roles in the progression and severity of pancreatitis.

#### Occludin

Occludin was the first transmembrane tight junction component identified. In a mouse model of AP, Schmitt et al., in 2004, showed that occludin in the apical membranes of intralobular duct cells and of acinar cells disrupted and disassembled within 5–10 min after intraperitoneal administration of supramaximal doses of caerulein [25]. This study revealed that occludin is degraded at a very early stage of AP, leading to disruption of tight junctions and increased paracellular permeability, which may augment interstitial edema formation. Sigrid et al. reported a molecular mechanism of occludin-dependent rearrangement of tight junctions [28]. Inhibition of Na-K-ATPase ion transport, which is localized to the apical junction, reduced PP2A activity, led to hyperphosphorylation of occludin, and induced rearrangement of tight junctions. This rearrangement resulted in increased permeability of tight junctions. Ethanol treatment of Capan-1 cell monolayers also increased paracellular permeability and downregulated occludin expression [29]. Xia et al. reported that administration of emodin (1,3,8-trihydroxy-6-methyl-anthraquinone), an anthraquinone derivative from the Chinese herb *Radix et Rhizoma Rhei*, which reportedly suppresses inflammatory cytokine production, promoted pancreatic occludin expression, reduced pancreatic paracellular permeability, which ameliorated pancreatic injury, and exerted a protective effect against experimental AP in rats [30]. Zymogen granules in acinar cells are exocytosed to the ductal lumen, inducing luminal acidification. This reduces occludin expression and disrupts tight junctions after supramaximal caerulein stimulation for isolated murine acinar cluster. Such disruption could be prevented by pH buffering [31]. Therefore, the exocytosis of zymogen granules may accelerate pancreatic damage during pancreatitis.

#### Tricellulin

Tricellulin, which is structurally similar to occludin, forms tricellular tight junctions at the contact point of three epithelial cells, which is required for the maintenance of the transepithelial barrier [32]. Tricellulin is localized to the apical side of the tricellular contacts of normal acini and ducts, while islets cells were negative [33]. Kojima et al. reported that the c-Jun N-terminal kinase pathway upregulates tricellulin expression in normal human pancreatic duct epithelial cells [34]. Tricellulin is overexpressed in well-differentiated pancreatic ductal adenocarcinomas but expressed weakly in poorly differentiated adenocarcinoma [33]. However, its role in pancreatitis is unclear.

#### Claudin

The claudin family, tight junction transmembrane components, has at least 24 members [19, 20]. Normal pancreatic duct and acinar structures express claudin-1, -2, -3, -4, -5, and -7 [35–37]. Schmitt et al. reported a discontinuous claudin-1 staining pattern in the apical membranes of intralobular duct cells, 38 and of acinar cells 5–10 min after supramaximal caerulein stimulation [25]. Claudin-2 was detected only in duct cells of the normal pancreas [36]; claudin-2 was also detected in acinar cells in a porcine model of AP, but claudin-3, -4, -5, or -7 immunoreactivity in acinar and duct cells did not differ before and after induction of AP [38]. Nakada et al. showed that claudin-4 and -7 mRNA levels were upregulated in mice with caerulein-induced AP and that acinar cells showed strong immunoreactivity for claudin-4. The upregulation of these genes could represent a response of acinar cells to repair and restore tight junctions [39]. Administration of emodin reportedly promotes pancreatic claudin-5 expression and exerts a protective effect in a rat model of AP [30].

#### Junctional adhesion molecules

The IgG-like family of JAMs is transmembrane components of tight junctions, and comprises JAM-A, -B, and -C. JAMs are found not only at tight junctions but also on the surface of leukocytes, thus contributing to their transendothelial migration [19]. In mice with caerulein-induced AP, the JAM-A mRNA level was upregulated [39], but its role in AP is unclear. Vonlaufen et al. showed that JAM-C is expressed in pancreatic blood vessels but not in acinar cells and ductal cells, and is upregulated in caerulein-induced AP [40]. Application of an anti-JAM-C antibody blocked leukocyte infiltration to the inflamed pancreas and reduced the severity of AP. Conversely, Wu et al. reported that JAM-C is downregulated in caerulein- and lipopolysaccharide-induced pancreatitis, and reduced JAM-C expression in AP was correlated with lung injury and increased reverse transendothelial migration of neutrophils [41]. The severity of AP injury did not differ significantly between JAM-C-deficient and wild-type mice, but JAM-C deficiency exacerbated lung injury and systemic inflammation. Thus, JAM-C downregulation may contribute to AP-associated acute lung injury by promoting reverse transendothelial migration of neutrophils [41].

#### Zonula occludins

ZOs, important cytoplasmic factors that bind to occludins, claudins, and JAMs, consist of ZO-1, -2, and -3 [19, 20]. Fallon et al. reported in 1995 that, in caerulein-hyperstimulated rats, ZO-1 staining in pancreatic ductal cells was clumped and disorganized after 30 min, and paracellular permeability was increased [26]. Schmitt et al. demonstrated a condensed and discontinuous irregular ZO-1 staining pattern in the apical membranes of intralobular duct cells and of acinar cells after 30 min of supramaximal stimulation with caerulein, following the early disassembly of occludin and claudin-1 [25]. Ethanol treatment of monolayers of Capan-1 cells downregulated ZO-1 expression [29]. The role of ZO-2 and -3 in pancreatitis is unclear.

### Adherens junctions

Adherens junctions are typically positioned below the tight junctions and function in tissue morphogenesis, initiation, and stabilization of cell–cell adhesion, homeostasis, and signal transduction [19, 20, 42, 43]. Epithelial adherens junctions are composed of the single-pass transmembrane protein, E-cadherin, as well as cytoplasmic p120-catenins, α-catenins, and β-catenins. The basic structure and function of these proteins were reviewed by Niessen [19] and Hartsock and Nelson [20]. The mechanisms of adherens junction dissociation and reassembly were examined by Lerch et al., who reported that the dissociation and internalization in acinar cells of adherens junctions occurred 2 h after caerulein injection, and that disruption of adherens junctions widened the interstitial space between adjacent acinar cells, resulting in interstitial edema [8]. The relocalization and reassembly of the major components of adherens junctions occurred 12 h after caerulein injection. They concluded that these dynamic changes of adherens junction structure are probably related to the mechanism of AP initiation and progression, and the rapid restoration of adherens junctions is likely to be the first step in the repair of acinar cells in AP [8]. Adherens junction organization is required for the assembly of tight junctions [44]; thus, adherens junctions are probably involved in pancreatic parenchyma reconstruction in pancreatitis. Schnekenburger et al. showed that adherens junctions are colocalized with, and maintained by, protein tyrosine phosphatases (PTPs) [45]. Tyrosine phosphorylation of the cadherin–catenin complex induced dissociation and internalization of the complex, and inhibition of PTPs resulted in dissociation of pancreatic adherens junctions [45]. Thus, tyrosine phosphorylation of the cadherin–catenin complex regulates cell contacts at adherens junctions, and receptor-type PTPκ and cytosolic PTP SHP-1 play important roles in the regulation, maintenance, and restoration of adherens junctions and cell contacts in pancreatic acinar cells [45, 46].

#### E-cadherin

E-cadherin is a Ca^2+^-dependent adhesion protein of the classical cadherin family. Schnekenburger et al. reported that the E-cadherin protein levels were not significantly affected by caerulein-induced AP in rats, but PTP-mediated internalization of E-cadherin–catenin complex was observed [46]. In contrast, Nakada et al. reported that the E-cadherin mRNA level was upregulated, and acinar cells showed strong immunoreactivity for E-cadherin in a mouse model of AP at 24 h after caerulein injection [39]. Yuan et al. reported that E-cadherin expression was significantly higher in rats with severe AP at 24 h after intraperitoneal injection of l-arginine [47]. Upregulation of E-cadherin expression is a protective response and promotes the repair of cell–cell adhesions of pancreatic acinar cells. Exocytosis of zymogen granules from acinar cells to the ductal lumen, the normal function of the pancreas, induced luminal acidification. The changes in extracellular pH caused by exocytosis of zymogen granules resulted in distribution of E-cadherin to the subapical region after supramaximal caerulein stimulation, which was ameliorated by pH buffering [31]. Therefore, exocytosis of zymogen granules may accelerate pancreatic damage in patients with pancreatitis.

Under the inflammatory conditions in rats with AP, extracellular cleavage of E-cadherin is induced by leukocyte elastase [48]. Dissociation of cell–cell contacts increases the transmigration of inflammatory cells into the pancreas during the initial phase of inflammation in experimental pancreatitis [48]. The product of degradation of the extracellular portion of E-cadherin is known as soluble E-cadherin (sE-cadherin). sE-cadherin was discovered as an 80 kDa peptide secreted by MCF-7 human breast carcinoma cells [49], and was detected by Katayama et al. in serum samples from healthy individuals and cancer patients [50]. The sE-cadherin levels were significantly higher in the serum of cancer patients than in that of the healthy individuals. Sewpaul et al. investigated the serum sE-cadherin levels in 19 mild AP and 7 severe AP patients at ≤ 12, 24, and 48 h after the onset of pain; the sE-cadherin level in the severe AP patients at ≤ 12 h was significantly higher than that in the mild AP patients [15]. That study was limited by its small sample size; thus, further research should examine the utility of the serum sE-cadherin level as a marker of the severity of AP.

#### p120 catenin

p120 catenin binds to the juxtamembrane region of the cytoplasmic portion of E-cadherin [51]. Leser et al. reported that tyrosine phosphorylation of p120 catenin occurred within 2 min after CCK stimulation of rat pancreatic acini [52]. CCK-dependent tyrosine phosphorylation of p120 catenin disrupted the F-actin cytoskeleton, and this effect was ameliorated by the tyrosine kinase inhibitor PP1 [52]. Schnekenburger et al. also demonstrated phosphorylation of p120 catenin in vivo after stimulation with supramaximal concentrations of caerulein [46]. Therefore, the phosphorylation of p120 catenin is associated with the disruption of adherens junctions in pancreatitis. A mouse model in which pancreas-specific p120 catenin was deleted was reported by Hendley et al. [53]. Conditional deletion of p120 catenin in the pancreas resulted in the formation of dilated epithelial tubules at the neonatal stage, and induced infiltration of CD45-positive inflammatory cells in pancreatic parenchyma, which resembled the pathology of CP. Interestingly, male mice with conditional p120 catenin deletion survived to adulthood, while female mice died during the early post-natal period, for unknown reasons. Therefore, the authors concluded that p120 catenin plays a crucial role in tubulogenesis and pancreatic development [53].

#### α-catenin

α-Catenin is a cytoplasmic component of adherens junctions and binds to β-catenin. α-Catenin also connects actin cytoskeleton [20, 51]. Schnekenburger et al. reported that the α-catenin protein level decreased in AP at 4 and 12 h after caerulein injection. This decrease in α-catenin level was not regulated by tyrosine phosphorylation [46]. The role of α-catenin in pancreatitis is unclear.

#### β-catenin

β-Catenin binds to the C-terminal cytoplasmic domain of E-cadherin and to α-catenin [20, 51]. β-catenin is not only a structural component of adherens junctions, but is also involved in the canonical Wnt/β-catenin pathway [20, 51], which plays multiple roles during embryonic development, including the development of the pancreas [54, 55]. Conditional knockout of β-catenin in the pancreas resulted in pancreatic hypoplasia, loss of exocrine pancreatic cells, increased abundance of duct-like structures, extensive fibrosis, and inflammatory cell infiltration; these changes resembled those in AP and CP [56]. In contrast, activated β-catenin gain-of-function in mice at an early embryonic stage (E10.5) resulted in disruption of the pancreas, near-total pancreatic agenesis, and formation of multiple large cysts. Interestingly, activated β-catenin gain-of-function in mice at a later embryonic stage (E11.5) resulted in an enlarged pancreas [55]. Keefe et al., by tamoxifen-inducible pancreatic-specific knockout of β-catenin, showed that β-catenin is required for the regeneration of pancreatic structure after caerulein-induced pancreatic damage [57]. Furthermore, loss of Notch signaling increased and prolonged β-catenin expression during exocrine regeneration after caerulein treatment, which impaired the regeneration of the pancreas after AP [58]. Taken together, these findings suggest that an appropriate level of expression of β-catenin is required for pancreatic maintenance and regeneration.

### Other junctions and adhesion molecules

The selectin family and ICAMs are expressed on the membrane of leukocytes and endothelial cells, and play roles in leukocyte adhesion and rolling in blood vessels [59]. These functions are important for induction of organ inflammation and disruption, not only in the pancreas parenchyma itself but also in distant organs, and are involved in the pathogenesis of systemic inflammation and multiple organ failure in severe AP.

#### Selectin

The selectin family comprises endothelial selectin (E-selectin), leukocyte selectin (L-selectin), and platelet selectin (P-selectin).

The levels of E-selectin and P-selectin are elevated in severe AP and, thus, may be biomarkers of AP severity [14, 60–63]; in contrast, the L-selectin level is not correlated with the severity of AP [14, 64, 65]. Elevated E- and/or P-selectin levels were correlated with a higher mortality rate, longer hospitalization, and development of pancreatic necrosis [60, 61]. Severe AP can lead to respiratory failure [pancreatitis-associated lung injury (PALI)], and the selectin family, particularly P-selectin, is involved in the progression of PALI [66–70]. An anti-P-selectin antibody prevented neutrophil infiltration in the pancreatic parenchyma, and ameliorated tissue inflammation and necrosis [71, 72]. Oxidative stress could increase P-selectin expression in lung, and xanthine oxidase inhibition could prevent the upregulation of P-selectin expression, the infiltration of neutrophils, and ameliorate the progression of PALI in rats [73]. Therefore, selectins are potential therapeutic targets in AP.

The serum sE-selectin and sP-selectin levels did not differ between post-ERCP pancreatitis (PEP) patients and healthy controls [74]. This may be because most PEP patients had mild disease.

E- and P-selectin are associated with the progression and severity of AP. However, plasma E-selectin levels did not differ between CP patients and healthy volunteers [75], possibly because of the relatively low severity of inflammation in CP.

#### ICAMs

ICAMs are important membrane glycoproteins that promote the attachment of cytokine-stimulated leukocytes to endothelial cells and initiate their transendothelial migration [76].

Serum ICAM-1 levels are elevated in AP, particularly severe and/or necrotizing AP [76–81]. An elevated serum ICAM-1 level was correlated with a higher mortality rate and development of pancreatic necrosis [78, 80] and, thus, is a potential early diagnostic and prognostic marker of severe AP. However, the serum ICAM-1 level could not be used to distinguish infected from sterile necrotizing pancreatitis [82], and an early change in the serum ICAM-1 level was not diagnostic of PEP [74]. Oxygen radicals and trypsinated serum are associated with the overexpression of ICAM-1 in experimental AP [9, 83]. Lack of inducible nitric oxide synthase significantly reduced ICAM-1 and P-selectin expression on the endothelial cells and correlated with reduction of leukocyte infiltration in mice model of pancreatitis [11]. Administration of an anti-ICAM-1 antibody to rats with AP significantly enhanced capillary blood flow in the pancreas, reduced leukocyte rolling, and stabilized capillary permeability [84]. Moreover, intracapillary leucocyte accumulation is reduced in ICAM-1 knockout mice with AP [85].

ICAMs are involved in the progression of PALI [66, 67, 86, 87]. Siemiatkowski et al. examined the levels of E-selectin, ICAM-1, tissue factor, and von Willebrand factor in the blood of severe AP patients with PALI and reported that an increased ICAM-1 level was predictive of PALI [66]. Application of an anti-ICAM-1 antibody ameliorated the lung injury in AP [86].

The plasma level of ICAM is reportedly significantly higher in CP patients than healthy subjects [75]; however, the role of ICAMs in the pathogenesis of CP is unknown.

## Concluding remarks

Adhesion molecules function in cell maintenance and homeostasis, as well as in the maintenance of tissue structure. Pancreatitis is typified by edema, disruption of pancreatic parenchyma, infiltration of inflammatory cells, and fibrosis; the normal pancreatic structure is almost destroyed. Maintaining the normal functions of adhesion molecules and preventing their abnormal activation would promote homeostasis and retention of a normal pancreatic structure. Further studies on the roles of adhesion molecules in the normal and injured pancreas will facilitate the development of novel therapeutic strategies against AP and CP.
